# Using electronic consultation (eConsult) to identify frailty in provider-to-provider communication: a feasibility and validation study

**DOI:** 10.1186/s12877-023-03870-w

**Published:** 2023-03-09

**Authors:** Ramtin Hakimjavadi, Sathya Karunananthan, Celeste Fung, Cheryl Levi, Mary Helmer-Smith, James LaPlante, Mohamed Gazarin, Arya Rahgozar, Amir Afkham, Erin Keely, Clare Liddy

**Affiliations:** 1 Faculty of Medicine, University of Ottawa, Ottawa, Canada; 2 C.T. Lamont Primary Health Care Research Centre, Bruyère Research Institute, Ottawa, Canada; 3 Interdisciplinary School of Health Sciences, University of Ottawa, Ottawa, Canada; 4 Department of Family Medicine, Faculty of Medicine, University of Ottawa, Ottawa, Canada; 5 St. Patrick’s Home of Ottawa, Ottawa, Canada; 6 Emergency Department Outreach Program, The Ottawa Hospital, Ottawa, Canada; 7 School of Population and Public Health, Centre for Health Services and Policy Research, University of British Columbia, Vancouver, British Columbia Canada; 8 Centre of Excellence for Rural Health and Education, Winchester District Memorial Hospital, Winchester, Ontario Canada; 9 Ontario Health East, Ottawa, Canada; 10 Ontario eConsult Centre of Excellence, The Ottawa Hospital, Ottawa, Canada; 11 Department of Medicine, University of Ottawa, Ottawa, Canada

**Keywords:** Frailty, Telemedicine, Electronic consultation, Identification, Unstructured data

## Abstract

**Background:**

Frailty is a complex age-related clinical condition that increases vulnerability to stressors. Early recognition of frailty is challenging. While primary care providers (PCPs) serve as the first point of contact for most older adults, convenient tools for identifying frailty in primary care are lacking. Electronic consultation (eConsult), a platform connecting PCPs to specialists, is a rich source of provider-to-provider communication data. Text-based patient descriptions on eConsult may provide opportunities for earlier identification of frailty. We sought to explore the feasibility and validity of identifying frailty status using eConsult data.

**Methods:**

eConsult cases closed in 2019 and submitted on behalf of long-term care (LTC) residents or community-dwelling older adults were sampled. A list of frailty-related terms was compiled through a review of the literature and consultation with experts. To identify frailty, eConsult text was parsed to measure the frequency of frailty-related terms. Feasibility of this approach was assessed by examining the availability of frailty-related terms in eConsult communication logs, and by asking clinicians to indicate whether they can assess likelihood of frailty by reviewing the cases. Construct validity was assessed by comparing the number of frailty-related terms in cases about LTC residents with those about community-dwelling older adults. Criterion validity was assessed by comparing clinicians' ratings of frailty to the frequency of frailty-related terms.

**Results:**

One hundred thirteen LTC and 112 community cases were included. Frailty-related terms identified per case averaged 4.55 ± 3.95 in LTC and 1.96 ± 2.68 in the community (*p* < .001). Clinicians consistently rated cases with ≥ 5 frailty-related terms as highly likely of living with frailty.

**Conclusions:**

The availability of frailty-related terms establishes the feasibility of using provider-to-provider communication on eConsult to identify patients with high likelihood of living with this condition. The higher average of frailty-related terms in LTC (versus community) cases, and agreement between clinician-provided frailty ratings and the frequency of frailty-related terms, support the validity of an eConsult-based approach to identifying frailty. There is potential for eConsult to be used as a case-finding tool in primary care for early recognition and proactive initiation of care processes for older patients living with frailty.

**Supplementary Information:**

The online version contains supplementary material available at 10.1186/s12877-023-03870-w.

## Introduction

Frailty is a health state characterized by a heightened vulnerability to poor recovery in the face of stress [1]. While not an inevitable part of aging, frailty results from an age-related decline in multiple physiological systems and is strongly associated with adverse outcomes including disability, morbidity, falls, hospitalisation, admission to long-term care, and mortality [2, 3]. However, over the course of a slowly progressive functional deterioration that typically spans five to ten years [4], there are opportunities to prevent these poor outcomes. Frailty is a dynamic, potentially reversible process that can be understood as a spectrum of intermediate states, with transitions between greater and lesser degrees of severity [1, 5]. For older adults living with frailty, early recognition and proactive intervention can help improve outcomes and potentially prevent, reduce, or delay further decline [6–8].

Identifying frailty is the first step towards improving clinical care for frail older adults [9]. Primary care has been proposed as the ideal setting for incorporating the concept of frailty and its identification into routine practice [10, 11]. Primary care providers (PCPs) are often the first point of contact for older adults, which enables earlier identification of patients who are at risk for or live with milder degrees of frailty [8]. Moreover, given their training, PCPs are predisposed to think about their patients from a more holistic viewpoint. This aligns closely with the concept of frailty, which is a practical, unifying understanding of vulnerability in the care of older adults that directs attention away from just the disease and towards the patients in whom it occurs [1, 11].

Any encounter between an older adult and their PCP is an opportunity to identify frailty and initiate appropriate care processes [12]. At such encounters, equipping PCPs with valid, reliable frailty identification tools can enhance clinical judgement and provide unrecognized opportunities for prevention, diagnosis, and care planning [11]. A number of tools for identifying frailty exist [13], but their adoption into routine clinical practice has not been entirely successful [14–16]. The use of formal screening instruments to detect frailty can be time-consuming or resource-intensive in the busy primary care clinic [17]. While simpler, clinician-oriented screening tools have been developed [18], such as the Edmonton Frail Scale [19], the perception that their use is time-consuming or disruptive to clinical workflows can lead to PCPs relying on rapid, intuition-based screening of frailty in their older patients instead [20]. The downside to this subjective approach is that many cases of frailty can be missed or overlooked [11, 20]. To help promote the consistent identification of frailty in primary care, helpful tools that are valid, are reliable, and help differentiate frailty status from normal aging are needed [17]. However, perhaps equally as important to ensure successful translation into the clinical setting, tools for detecting frailty must be simple and clinically sensible as well [21, 22].

In the face of these challenges for developing a suitable tool for PCPs to adopt and use in routine practice, some authors have suggested the use of existing clinical datasets to measure and detect frailty [1, 23]. In recent years, there has been a growing interest in leveraging healthcare databases to identify frailty [24]. Frailty definitions based on electronic medical record (EMR) data and health administrative databases have found varying degrees of success [24–28]. A common theme has been that efforts to learn about frailty using healthcare data face challenges that are inherent to the limitations of the datasets being examined [24]. For example, cognitive and functional impairments, information-rich components of frailty [2], are often poorly coded or altogether missing in hospital-based datasets [28, 29]. As a result, many administrative or EMR-based definitions of frailty may be underrepresenting the true prevalence of frailty [25, 30].

One source of healthcare data that has been relatively unexplored is provider-to-provider communication, such as that found in telemedicine-derived data [31]. The increasing use of telemedicine tools presents a unique opportunity to examine clinicians’ descriptions of their patients and may provide a source of data that captures additional information about a patient’s overall health state.

Electronic consultation (eConsult) is one such telemedicine tool that captures provider-to-provider communication. eConsult is a web-based primary care tool that enables PCPs to submit a patient-specific question to a specialty group of their choosing; directly communicate with a specialist through a secure platform; and receive timely advice concerning their patient’s care [32]. eConsult is already being used by PCPs caring for older adults in the community and LTC [33–35]. Provider-to-provider communication captured on eConsult is a potentially rich source of information that can reveal patient-centred insights. By harnessing the text-based data in eConsult communication, it may be possible to use providers’ descriptions of patients to identify frailty and leverage eConsult as an initial case-finding tool in primary care. In what has been proposed as a two-step approach for frailty assessment [36], identified cases of an at-risk population could then lead to more complex or time-consuming frailty assessment tools, followed by comprehensive care planning and personalized interventions for frailty.

## Aims and objectives

The aim of the study was to explore the feasibility and validity of identifying frailty status using provider-to-provider communication captured in eConsult cases. We hypothesized that because there is a higher prevalence of frailty in LTC [37], PCPs’ descriptions of LTC residents would have a higher frequency of frailty-related terms. We further relied on literature support and clinician expertise to develop an operational definition of frailty on eConsult. Using this as our basis, we extracted the text from eConsult cases and searched for frailty-related terms. Guided by Rockwood’s proposal for validation of a successful definition of frailty [21], and the theory for validity in quantitative studies [38, 39], we sought to establish the content, construct, and criterion validity of our approach by answering the following research questions:

1. Is there sufficient information captured in an eConsult interaction to make inferences about the frailty status of the patient being described?
2. Are frailty-related terms more frequently used in eConsult cases about residents living in LTC compared to those about older adults living in the community?
3. Is there agreement between the frequency of frailty-related terms identified in eConsult text and clinician-provided frailty ratings of eConsult cases based on clinical judgement?

## Methods

This study was a retrospective analysis comparing eConsult cases submitted on behalf of LTC residents (“LTC cases”) versus cases submitted on behalf of community-dwelling older adults (“community cases”), using a 1:1 matching design based on patient age and gender. Cases were characterized by 1) a text-parsing computer algorithm to identify frailty-related terms in the text of communication logs, and 2) a clinician-led review of cases to provide clinical judgement of the level of frailty-related content in the eConsult. Study conception, design, and interpretation of findings were conducted with a multidisciplinary team (see "Patient and Public Involvement” in Supplemental Methods).

### Setting

The Champlain BASE™ eConsult service operates in the Champlain region – a health region located in Eastern Ontario, Canada with a population of 1.3 million, of which more than 250,000 are aged 65 years or older. Once registered on the eConsult service, PCPs may submit a non-urgent patient-specific clinical question to one of 150 specialty and sub-specialty groups, attaching any additional files they deem relevant to the case (e.g., additional notes, imaging reports, lab results). Each case is assigned to a specialist based on their availability, and specialists are asked to reply within seven days. In responding, specialists can do any of the following: provide a recommendation, request more information, or recommend a face-to-face referral. The service allows PCPs and specialists to engage in iterative, asynchronous communication. The discussion can occur until the PCP ultimately decides to close the case.

### Data collection

Eligible cases were those concerning a LTC resident or an age- and gender-matched community-dwelling older adult, submitted between January 1, 2019, and December 31, 2019. We identified LTC cases as those submitted by physicians or nurse practitioners who, upon registering with the service, indicated a LTC facility as their practice address. Community cases were selected from cases submitted by physicians or nurse practitioners who were not affiliated with a LTC home. One hundred and fourteen LTC cases were first randomly sampled, and community cases were then matched based on patient age and gender. LTC residents younger than 55 years were matched to community-dwelling patients aged 55 ± 1 year. Although an age threshold for older adults is commonly set at 65, in many cases frailty onset starts before this age [1, 40]. We identified eConsult cases about older adults using a lower threshold to reflect this.

A completed eConsult case includes the initial communication by the referring PCP (including clinical question(s) posed), the response(s)/advice from a specialist, and any further exchange between the providers. Cases with missing communication logs and those containing communication in French were excluded.

Basic service utilization data is collected from all eConsult cases. For the present study, the following utilization data were collected from each case: the patient’s age and gender, the specialist response time and the specialists’ self-reported amount of time billed.

### Clinician-led review of frailty content on eConsult

Two LTC clinicians (CF, C. Levi) reviewed the eConsult cases to provide their judgement of the likelihood that a given case was about a patient living with frailty. The reviewers were blinded to the settings to which the cases belonged (i.e., LTC or community).

Clinicians provided a judgement based on their clinical intuition and subjective interpretation of the case’s purpose and context. They were instructed to make these judgements (henceforth referred to as “frailty ratings”) according to a 5-point Likert scale, where a minimum score of ‘1’ indicates a very low likelihood that an eConsult case was about a patient living with frailty, and a maximum score of ‘5’ indicates a very high likelihood. Clinicians provided two frailty ratings per case: one after reading only the PCP’s initial communication (“frailty rating 1”), and another after reading the full eConsult interaction between the PCP and specialist (“frailty rating 2”). In relation to the first research question, we hypothesized that there would often be sufficient information captured in the PCP’s initial communication (corresponding to frailty rating 1) to make inferences about the frailty status of the patient being described. Details on the development and piloting of the frailty rating task are provided in the corresponding section in Supplemental Methods.

After independently reviewing the first 20 cases in the sample, the clinicians met to discuss their findings and resolve any discrepancies in their approach. Once consensus was achieved between reviewers by re-reviewing and discussing the case communication logs, coding for the remaining cases was split evenly (i.e., each classified by a single reviewer).

### Developing a frailty identification approach using eConsult

The approach to identifying frailty-related terms on eConsult was developed in two phases:

1. Creating a list of frailty-related terms based on a literature search and expert consultation.
2. Developing a computer algorithm to automatically parse eConsult text to search for frailty-related terms.

### Phase 1 – Preparing a key-term search to identify frailty content in eConsult

We performed a focused literature search and consulted with a working group of experienced clinicians, researchers, and patient partners to collate a list of frailty-related terms. This approach was adapted from Urquhart et al.’s development of a rule to identify frailty in administrative health databases [25]. The aim was to construct a list of terms or phrases that would commonly be used by providers to describe patients living with frailty.

The focused literature review involved an initial scan of PubMed using combinations of the following terms: “frail elderly”, “frailty”, “identification”, “definition”, “database”, and “health data”. We were particularly interested in studies that have used key-term searching to identify frailty in the free text of other healthcare datasets. We considered a variety of study types, including systematic reviews and other evidence syntheses, clinical guidelines, retrospective studies of healthcare or administrative databases, and studies that have developed or validated frailty assessment tools. Search results were supplemented by articles recommended by the research team and a hand search of reference lists of selected articles.

Relevant findings from the literature search were summarized. From each included study, we extracted terms related to the identification or assessment of frailty, including but not limited to signs and symptoms, comorbidities, disabilities, and related clinical syndromes.

To ensure the content validity of our selection, the preliminary list of frailty-related terms was then shared for feedback with a working group of clinicians (*n* = 4), researchers (*n* = 4) and a patient partner (*n* = 1) who are knowledgeable about LTC, primary care and frailty, each bringing diverse perspectives on these topics (see corresponding section in Supplemental Methods). Through iterative discussions and revisions, a version of the list of frailty terms was finalized for a key-term search of the eConsult text. The list was organized by grouping terms into overarching topic categories.

### Phase 2 – Developing an eConsult text-searching computer algorithm

A computer algorithm was developed using the Python programming language to parse eConsult text and perform a search for the frailty-related terms collated in Phase 1 (see corresponding section in Supplemental Methods). As input, the program is fed the list of frailty-related terms and the source text (eConsult communication logs) to parse. The list of frailty-related terms and the eConsult text were first cleaned and made suitable for use in the program. Using the formatted input data, the program parses the text of each eConsult case and records each time one of the key terms is encountered. As output, the program provides 1) the overall word count and, 2) the frequency that each frailty-related term appears within the text for each eConsult case. The Python code used in this analysis is available from the authors upon reasonable request.

### Validation and statistical analysis

To address the first research question (“*Is there sufficient information captured in an eConsult interaction to make inferences about the frailty status of the patient being described?*”), descriptive statistics for applying the text-parsing program were calculated to assess the availability of frailty-related terms captured on eConsult, and the proportion of cases deemed by clinicians to have sufficient information to assign a frailty rating was also assessed.

To establish construct validity and address our second research question (“*How frequently are frailty-related terms used in eConsult cases about residents living in long-term care compared to those about older adults living in the community?*”), we compared the frequency of frailty-related terms in LTC cases and community cases. This comparison served to assess whether the number of frailty-related terms observed in the eConsult communication coheres with other measures of the phenomenon (i.e., being a LTC resident) [21]. The overall word count per case was examined alongside the frequency of frailty-related terms to assess whether a greater incidence of frailty-related terms is not simply a function of a larger total number of words (i.e., a longer eConsult communication).

To establish criterion validity and address our third research question ("*Is there agreement between the frequency of frailty-related terms identified in eConsult text and clinician-provided frailty ratings of eConsult cases based on clinical judgement?*”), we graphically plotted the number of frailty-related terms identified in the eConsult communication logs against the clinician-provided frailty ratings. Here, the goal was to assess whether there was agreement between the frequency of frailty-related terms in the eConsult text and a ‘gold standard’ assessment of frailty [21], that is, clinicians’ judgement of a patient’s frailty status.

For comparisons, we used chi-squared tests for categorical variables or Student’s t-tests for continuous variables when examining for statistically significant differences. Statistical significance was defined with the threshold (α) of 0.05. Continuous variables were presented as means and standard deviations (SDs), and discrete variables as frequencies and percentages. Analysis was performed in Microsoft Excel (2016).

### Research Ethics approval

The Ottawa Health Science Network Research Ethics Board provided ethics approval for this study (Protocol #2009848-01H). Research Ethics approval was for the secondary data analysis. No written or verbal consent from participants was obtained.

## Results

### List of Frailty-related Terms

The focused literature search yielded population-based studies of healthcare databases [27, 30, 41] and clinical practice guidelines for frailty [42, 43] that informed the collation of a list of frailty-related terms. Based on consensus judgement from an expert panel, Anzaldi et al. (2017) developed a list of phrases for ten geriatric syndromes to be searched for in the free text of electronic health records [27]. We expanded upon the ten geriatric syndromes to include a total of seventeen topics relevant for identifying frailty in eConsult text (Table 1). An expanded list of related terms was generated for each topic (Table S1).

**Table 1:** Overall prevalence of frailty-related terms identified in the complete eConsult communication between PCP and specialist across all cases (*n* = 225)

**Frailty topic**	**Prevalence of terms (Total [LTC, Community])**^a^	**Most frequent terms in descending order**
Dementia^b^	165 (122, 43)	“dementia”, “cognitive impairment”, “delirium”, “of dementia”, “with dementia”, “moderate dementia”, “and dementia”, “has dementia”, “for dementia”, “alzheimer’s dementia”
Walking difficulty^b^	123 (107, 16)	“assistance”, “wheelchair”, “wheelchair bound”, “walker”, “wheelchair dependent”, “in a wheelchair”, “ataxia”, “impaired balance”, “paraplegia”, “is wheelchair bound”
Falls^b^	91 (58, 33)	“falls”, “fall”, “fell”, “a fall”, “recent fall”, “multiple falls”, “lost weight”, “had a fall”, “recent falls”, “collapse”
Frailty syndrome^c^	78 (43, 35)	“failure”, “obstruction”, “frail”, “cervical”, “frailty”, “metabolism”, “is frail”, “vulnerable”, “deficit”, “fragile”
Clinical Frailty Scale^c^	77 (62, 15)	“dependent”, “end-stage”, “vulnerable”, “dependence”, “ADL”, “mildly frail”, “IADL”
Other medical concepts^c^	56 (34, 22)	“osteoporosis”, “constipation”, “chronic pain”
Psychological^c^	38 (29, 9)	“depression”
Weight loss^b^	24 (11, 13)	“weight loss”, “emeron”, “lost weight”, “progressive weight loss”, “losing weight”, “poor appetite”, “lost wt”, “not eating”, “decreased appetite”
Lack of social support^b^	18 (10, 8)	“alone”, “social worker”, “no family”, “cannot afford”, “financial assistance”, “case management”
Functional capacity^c^	18 (6, 12)	“fatigue”, “disability”, “impaired balance”, “atrophy”, “malaise”, “loss of energy”
Clinical assessment of frailty^c^	14 (5, 9)	“MoCA”, “Clinical frailty scale”, “medication review”
Health service utilization^c^	14 (12, 2)	“palliative care”, “goals of care”, “hospice”
Severe urinary control issues^b^	7 (7, 0)	“indwelling catheter”, “chronic indwelling catheter”
Visual impairment^b^	7 (4, 3)	“can’t see”, “blind”, “legally blind”
Absence of fecal control^b^	3 (3, 0)	“fecal incontinence”, “incontinent of stool”
Pressure ulcers^b^	2 (2, 0)	“pressure ulcer”, “sacral ulcer”
Malnutrition^b^	0 (0, 0)	-

*Abbreviations*: *LTC* long-term care

^a^Sample sizes: Total *n* = 225 eConsult cases, LTC *n* = 113 cases, Community *n* = 112 cases

^b^Frailty topics derived from Anzaldi et al.’s ten geriatric syndromes

^c^Additional frailty topics added through a focused literature search and expert consultation

### eConsult sample

We sampled 114 eConsult cases about residents in LTC and 114 cases about older adults in the community, submitted by 114 unique PCPs. There were no cases with missing communication logs in the sample. There was one duplicate case and two cases in French, resulting in three cases being excluded. After removing these cases, 113 LTC cases and 112 community cases met our eligibility criteria and were included for analysis. The characteristics of included cases, stratified by setting, are provided in Table 2.

**Table 2:** Characteristics of Long-term care and Community eConsult cases included for analysis

**a.** Total sample		
**Setting**	**LTC (mean** ± **SD)**	**Community (mean** ± **SD)**
**N**	113	112
**Patient age**	80.6 ± 11.6	79.8 ± 11.2
**Patient gender (% female)**	62.8%	62.5%
**Overall word count per case**	586.22 ± 393.54	458.62 ± 362.74
**Frequency of frailty-related terms per case**	4.55 ± 3.95^c^	1.96 ± 2.68^c^
**Cases with ≥ 1 frailty-related term(s) (n, %)**	105, 92.9%^c^	68, 60.7%^c^
**Cases with highest rating for Frailty rating 1**^**a**^** (n, %)**	107, 96.7%	55, 55.6%
**Cases with highest rating for Frailty rating 2**^**a**^** (n, %)**	106, 95.5%	52, 57.8%
**b**. Subset of cases deemed to have insufficient information to make a frailty rating^b^		
**N**	2	22
**Overall word count per case**	363.00 ± 56.57	322.86 ± 146.83
**Frequency of frailty-related terms per case**	1.00 ± 0.00	0.32 ± 0.78

*Abbreviations*: *LTC* long-term care, *SD* standard deviation

^a^Frailty ratings were assigned for each case by clinicians. Frailty rating 1 represents clinicians’ assessment after reading the initial PCP communication only, and frailty rating 2 represents their assessment after reading the full PCP-specialist eConsult communication

^b^eConsult cases were deemed to have insufficient information by clinicians when attempting to assign a frailty rating

^c^The difference between LTC cases and community cases was statistically different (*p* < .001)

### Prevalence of frailty-related terms

The text-parsing algorithm was applied to the complete eConsult communication log for all 225 cases in the sample. After searching for all terms under each of the 17 frailty-related topics that we compiled in Phase 1 (Table S1), the only topic for which none of its terms were identified in the text was Malnutrition (Table 1). For most frailty-related topics (13 of 17), there was a higher frequency of frailty-related terms in the eConsult text of LTC cases compared to community cases (Table 1).

There was a significantly higher mean number of frailty-related terms per LTC case (4.55 ± 3.95) compared to community cases (1.96 ± 2.68; *p *< 0.001) (Table 2a). There was also a significantly higher proportion of LTC cases with at least one frailty-related term (92.9%, *n *= 105) compared to community cases (60.7%, *n* = 68; *p* < 0.001). For the range between 0 to 10 frailty-related terms, the mean overall word count per case increased by a factor of less than two: the mean and SD for cases with 0 and 10 frailty-related terms was 378.33 ± 442.19 and 613.25 ± 125.54 words, respectively (Table S2, Figure S1).

**Fig. 1:**
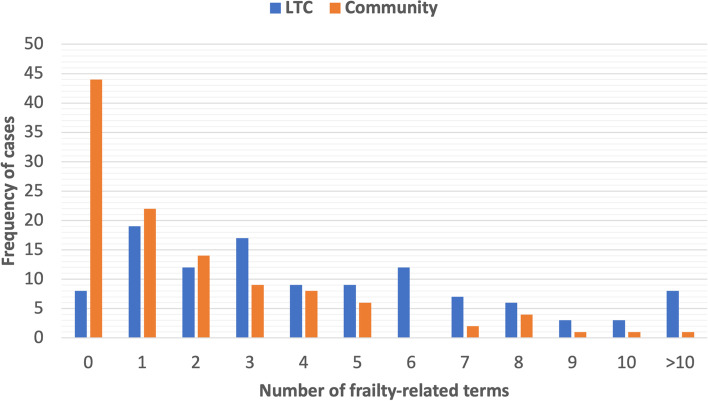
Distribution of long-term care cases and community cases by number of frailty-related terms in the complete PCP-specialist eConsult communication. LTC, long-term care

Figure 1 displays the distribution of LTC cases and community cases by the number of frailty-related terms identified in the text. Compared to LTC cases, there were many more community cases that had fewer than three frailty terms. Conversely, cases with three or more terms were more frequently in LTC rather than community-dwelling.

### Clinician-provided frailty-ratings

Clinicians (CF, C. Levi) reviewed the 225 cases in the sample to assign two frailty ratings per case. In two LTC cases (1.8% of *n *= 113) and 22 community cases (19.6% of *n* = 112), clinicians deemed there was insufficient information to assign such ratings (Table 2b). Thus, in 89.3% of 225 cases in the overall sample, there was sufficient information in the eConsult communication log to assess frailty status. Subsequent analyses of the frailty ratings were based on these 201 cases (111 LTC and 90 community cases).

**Fig. 2:**
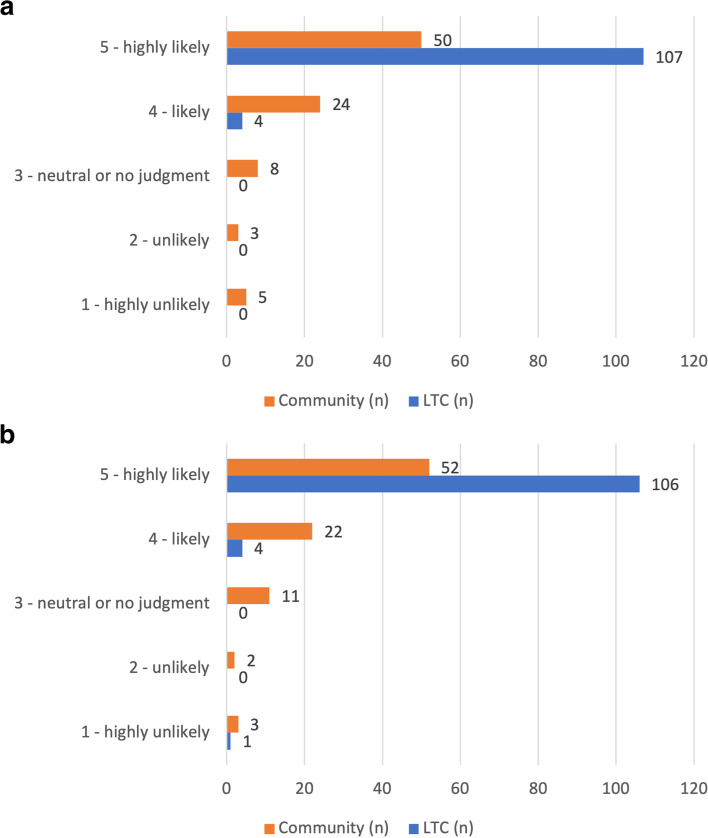
Clinician-provided frailty ratings. For each eConsult case, clinicians provided two frailty ratings to assign a likelihood that the patient described in the communication logs is living with frailty. A greater rating corresponds with a higher likelihood of living with frailty. **a** Clinician-provided frailty ratings after reading only the PCP’s initial communication in the submitted eConsult. LTC, long-term care; PCP, primary care provider. **b** Clinician-provided frailty ratings for frailty after reading the entire PCP-specialist eConsult interaction. LTC, long-term care

The distribution of both frailty ratings for the remaining 201 cases is provided in Fig. 2, demonstrating that all ratings were highly skewed towards a rating of 5. For each case, clinicians read only the initial PCP communication to provide frailty rating 1 (Fig. 2a) and then read the entire PCP-specialty interaction to provide frailty rating 2 (Fig. 2b). Overall, only eight ratings (4.0%) changed between the first and second assessment: one LTC case and seven community cases. Frailty ratings for the sole LTC case decreased by 4 (i.e., from a frailty rating of 5 to 1). For the community cases, five ratings increased (median increase of one frailty rating point) and one rating decreased (by one point). Community cases had a higher proportion of ratings less than 5 (i.e., 44.4% of 90 cases received a rating of 4 or less for frailty rating 1) compared to LTC cases (i.e., 3.6% of 111 cases received a rating of 4 or less for frailty rating 1). A similar pattern was observed for frailty rating 2 (Fig. 2b).

### Agreement between frequency of frailty-related terms and clinician-provided frailty ratings

**Fig. 3:**
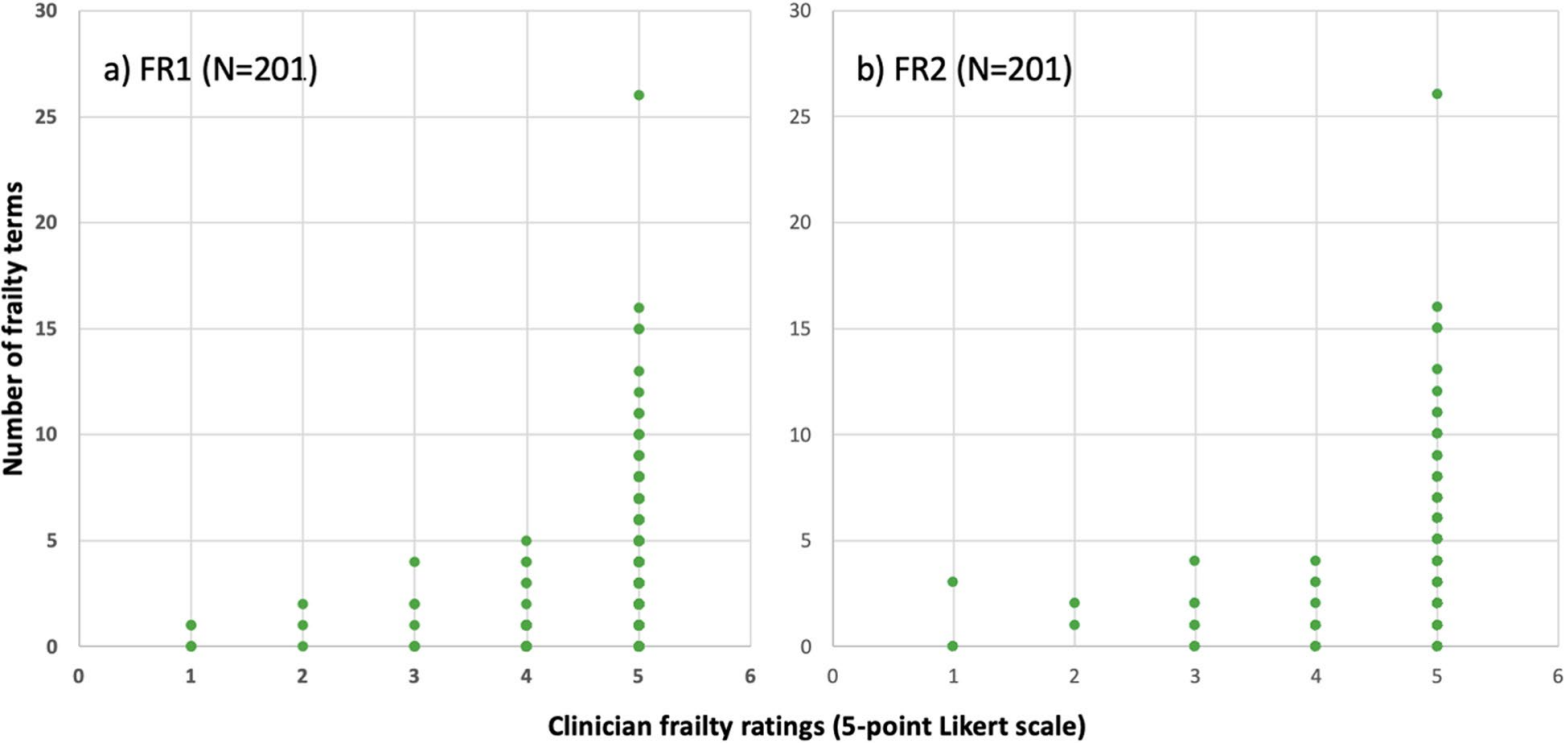
Plot of the clinician-provided frailty ratings against the number of frailty-related terms identified in the eConsult text. FR, frailty rating

The number of frailty-related terms per case was plotted against the clinician-provided frailty rating for frailty rating 1 (Fig. 3a) and frailty rating 2 (Fig. 3b). It was observed that for both frailty ratings, eConsult cases with greater than five frailty-related terms were assigned a clinician rating of five (i.e., a high likelihood that the patient being described is living with frailty). The total word count per case was also plotted against the clinician-provided frailty rating for frailty rating 1 (Figure S2a) and frailty rating 2 (Figure S2b).

Based on the distribution in Fig. 3, we selected five frailty-related terms as the cut-off for a binary classification of frailty in our eConsult sample. That is, cases with ≥ 5 frailty-related terms were classified as highly likely of living with frailty. When the classification was applied, 63 cases (28.0% of total sample; 48 LTC cases, 15 community cases) were classified as highly likely of living with frailty.

## Discussion

In this study, we explored the feasibility of identifying frailty status using provider-to-provider communication on eConsult. To our knowledge, this is the first study examining unstructured telemedicine-derived data to identify frailty. Our approach involved measuring the frequency of a list of pre-selected, expert-derived frailty-related terms in the text of eConsult communication logs to make inferences about whether the patient being described was living with frailty or not. Based on a review of the cases by clinicians experienced in frailty care, we further examined the frailty-related content by applying two Likert-type frailty ratings to each case. Although all ratings were highly skewed to a rating of 5 (Fig. 2), indicating a high likelihood that the patient described is living with any degree of frailty, we observed a trend whereby cases with 5 or more frailty-related terms corresponded to a higher frailty rating (Fig. 3). As a first step in using our frailty identification method, we applied a threshold (≥ 5 frailty terms) to classify cases as “frail” or “not frail”. Given the exploratory nature of this analysis, further refinement of the strategy to identify frailty and the selection of optimal cut-off values is a subject of further investigation by our group.

Overall, our findings support that there is richness in provider-to-provider communication captured on eConsult and sufficient information therein to make inferences about the frailty status of the patient being described. Terms related to 16 of 17 frailty-related topics were identified when searching across all eConsult cases in the sample (Table 1). Moreover, upon review of cases by experienced clinicians, only a small proportion of cases were flagged as having insufficient information to making a frailty rating (1.8% of LTC cases, and 19.6% of community cases). This finding is significant given frequent challenges reported in analysing structured healthcare datasets to identify frailty (e.g., administrative claims data, diagnostic codes in electronic medical records). It has been reported by other authors [27, 30], and in a recent review [24], that certain concepts (e.g., cognition, functional impairment) may not be captured in traditional healthcare datasets [29]. A study by Kharrazi et al. demonstrated that natural language processing, a text-mining technique, applied to unstructured data in clinical notes can significantly improve the detection of dementia, falls, malnutrition, and lack of social support [44]. These are geriatric syndromes that were also included in our list of frailty-related terms (Table 1). Our findings suggest that eConsult-based research may offer an opportunity to extend on the work by Kharrazi et al., i.e., as another data source for applying natural language processing to support enhanced identification of frailty. Interestingly, there were few intra-case changes between the first frailty rating based on the initial PCP communication and the second frailty based on the full eConsult interaction (changing in eight cases overall), supporting our hypothesis that there may be sufficient information in the PCPs’ initial description of the patient to make inferences about frailty status.

To assess the content validity of our proposed frailty identification method, we consulted the peer-reviewed literature and experienced clinicians to select a set of terms that adequately covers the multidimensional nature of frailty [1, 21]. Our operational definition of frailty aligns with the class of definitions that define frailty on the basis of a geriatric syndrome, such as delirium and falls [45, 46]. We posited that a greater number of terms related to one of the geriatric syndromes in eConsult communication logs would correspond with a greater likelihood of the patient being described is living with frailty. Drawing from methods originally developed by Anzaldi et al. [27], and through discussion with a working group of clinicians, researchers and patient partners, we expanded on the original ten geriatric syndromes by adding seven additional topics deemed to hold significance for identifying frailty (Table 1).

We evaluated construct validity by comparing the frequency of frailty-related terms between eConsult cases submitted from LTC and those from the community, hypothesizing that a greater frequency of terms would be observed in LTC cases given literature support for the higher prevalence of frailty in this setting [37, 47]. There was a significantly higher mean frequency of frailty-related terms in LTC cases compared to community cases (Table 2). Moreover, it did not appear that a higher frequency of frailty-related terms could be solely explained by a greater overall word count in the eConsult communication log (Table S2, Figure S1). This supports our hypothesis that eConsult cases submitted on behalf LTC residents are more likely to contain frailty-related language and provides evidence of construct validity for identifying patient frailty status in eConsult communications.

Our approach to evaluating criterion validity involved assessing whether there was agreement between our operational definition of frailty and an accepted ‘gold standard’ definition of frailty – in this context, clinical judgement of frailty-related content provided by experienced LTC clinicians. There was overall agreement between the number of frailty-related terms identified in the eConsult text and the clinician-provided frailty ratings, as observed by the positive trend depicted in Fig. 3. This supports the criterion validity of measuring the frequency of frailty-related terms to identify frailty on eConsult, as the frequency of such terms was in alignment with expert opinion regarding frailty status. While this initial analysis indicated that the presence of five or more frailty-related terms was highly consistent with clinician ratings of high likelihood of frailty, it should be noted that there were several cases where fewer than five frailty-related terms were rated by clinicians as highly likely of being frail. A cut-off of five terms may therefore provide a highly sensitive measure of frailty, but the specificity of this cut-off may be limited—that is, several patients living with frailty may in fact be missed. Future analyses should therefore take into consideration the specific terms in addition to the count to improve the specificity of the proposed methods. Application of a Receiver Operating Characteristic (ROC) graph with a widely accepted gold standard may also provide useful insights into the appropriate cut-offs when using eConsult data to identify frailty.eConsult is a tool that is used in primary care, and its utility for improving access to specialist advice for older patients has been demonstrated [33, 34, 48]. Primary care has been proposed as the ideal place to proactively screen for and identify frailty [17, 49, 50], particularly given the relatively frequent presentation of older adults to their PCPs [51]. Recently, the World Health Organization has called for healthcare professionals and policy-makers to look beyond disease states and move towards a more holistic approach to older adult care, one in which the main goals become the prevention of declines in “intrinsic capacity” and the maintenance of “functional ability” [52]. PCPs’ role in patient care naturally aligns with this, given their training and predisposition towards focusing on the individual as a whole and not just about their diseases [10]. For this reason, further research on the potential role of eConsult as an active case-finding tool [42, 52] for the proactive identification of older people in the community at risk of frailty is warranted, which can provide opportunities for social and health interventions before the onset of decline.

### Limitations

Our study has several limitations. First, there are inherent challenges to identifying frailty through eConsult data alone. An eConsult case is a snapshot in time that illustrates one component of a patient’s medical journey, and it is at the submitting PCP’s discretion to include whatever information that is deemed relevant for communicating their clinical question. This emphasizes the importance of using eConsult-based frailty identification less as a label (e.g., labelling a patient as “frail” or “not frail”) and more as an opportunity for a holistic discussion around care needs and the support and services required to meet the needs of the patient. Each frailty-related term or topic captured through provider-to-provider communication represents an opportunity to recommend resources or services specifically tailored to what was detected in the PCP’s eConsult communication. Second, we did not grade the degree of frailty. Frailty is a dynamic and potentially reversible state [22], thus future work should avoid simplistic binary classifications and instead look to examine degrees of frailty based on a continuum of features identified in the data. Third, frailty was identified based on PCP descriptions, which are dependent on perceptions and attitudes that can vary between clinicians, particularly given the complex and emerging nature of the frailty concept [20, 53]. For example, if PCPs lack knowledge of what contributes to frailty, they may not include these terms in their communication and thus the patient would continue to go unidentified. Fourth, we did not establish criterion validity by comparing our eConsult-derived definition of frailty to a gold standard instrument. However, given the limitations of our dataset with respect to patients’ health information, limited assessments beyond the descriptions of the eConsult user (i.e., the PCP or specialist) could be performed. Instead, our study enlisted the judgement of two experienced PCPs in frailty care to rate the frailty-related content in eConsult communications. Future studies may assess predictive validity as an alternative, by linking eConsult cases to health administrative data to evaluate our frailty definition by its ability to predict risk of adverse outcomes [21]. Finally, eConsult cases were identified as being submitted on behalf of LTC residents based on the primary organization of the PCP registered with the service. Because LTC clinicians may be managing some patients who do not or no longer reside in LTC, it is not possible to ensure complete accuracy in distinguishing between LTC and community-dwelling patients in our dataset.

## Conclusions

In conclusion, provider-to-provider communication captured on eConsult contains an unharnessed source of rich text data that can reveal insights about the frailty status of the patient being described by clinicians using the platform. We established the validity of an approach that measures the frequency of frailty-related terms in the text of eConsult communication logs to make inferences about whether the patient being described is living with frailty or not. This supports the feasibility of using eConsult as a case-finding tool for frailty in primary care, warranting further investigation of optimal implementation strategies for this approach in routine clinical practice. Future work will involve using eConsult-based frailty identification as an opportunity for making automatic recommendations to PCPs for evidence-based interventions or local resources to support integrated, community-based care. Given the challenges PCPs face with identifying frailty [10, 20], the present study is an important step towards equipping PCPs with a tool to proactively recognize and initiate appropriate care processes for their older patients living with frailty.

## Supplementary Information

**Additional file 1: Supplemental Methods:** a description of the methods for patient and public involvement, clinician-led review of frailty related content on eConsult, and phase 1-2 of developing a frailty identification approach using eConsult. **Supplemental Table S1.** List of frailty-related terms for each frailty topic. List of frailty-related terms for each of the 17 topics. **Supplemental Table S2.** Prevalence of eConsult cases stratified by number of frailty-related terms. Number of cases and mean overall word count per eConsult case, stratified by the number of frailty-related terms identified in the complete eConsult communication logs. **Supplemental Figure S1.** Mean overall word count per eConsult case, stratified by the number of frailty-related terms identified in the complete eConsult communication log. **Supplemental Figure S2.** Plot of the clinician-provided frailty ratings against the total word count in the eConsult text.

## Data Availability

The datasets generated and/or analysed during the current study are not publicly available because they contain confidential patient identifiable information. Data that do not include patient identifiable information are available from the corresponding author on reasonable request.

## Abbreviations

eConsult: Electronic consultation
LTC: Long-term care
PCP: Primary care provider
SD: Standard deviation
