# Supplementary material for: Using electronic consultation (eConsult) to identify frailty in provider-to-provider communication: a feasibility and validation study
Source: BMC Geriatr. 2023 Mar 9;23:136. doi: 10.1186/s12877-023-03870-w (PMC9999527; doi:10.1186/s12877-023-03870-w)
Supplement: Supplementary file 1 — Additional file 1: Supplemental Methods: a description of the methods for patient and public involvement, clinician-led review of frailty related content on eConsult, and phase 1-2 of developing a frailty identification approach using eConsult. Supplemental Table S1. List of frailty-related terms for each frailty topic. List of frailty-related terms for each of the 17 topics. Supplemental Table S2. Prevalence of eConsult cases stratified by number of frailty-related terms. Number of cases and mean overall word count per eConsult case, stratified by the number of frailty-related terms identified in the complete eConsult communication logs. Supplemental Figure S1. Mean overall word count per eConsult case, stratified by the number of frailty-related terms identified in the complete eConsult communication log. Supplemental Figure S2. Plot of the clinician-provided frailty ratings against the total word count in the eConsult text. [file 12877_2023_3870_MOESM1_ESM.zip › BMC Supplementary materials - 16Sept22.docx]

**Legend**

Supplemental Methods (“BMC Supplementary materials.docx”; page 2): a description of the methods for patient and public involvement, clinician-led review of frailty related content on eConsult, and phase 1-2 of developing a frailty identification approach using eConsult.

Supplemental Table S1. List of frailty-related terms for each frailty topic. (File name: “Supplementary Table S1.xlsx”): list of frailty-related terms for each of the 17 topics.

Supplemental Table S2. Prevalence of eConsult cases stratified by number of frailty-related terms (File name: “BMC Supplementary materials.docx”; page 3): Number of cases and mean overall word count per eConsult case, stratified by the number of frailty-related terms identified in the complete eConsult communication logs.

Supplemental Figure S1 (File name: “Supplemental Figure 1.png”): Mean overall word count per eConsult case, stratified by the number of frailty-related terms identified in the complete eConsult communication log.

Supplemental Figure S2 (File name: “Supplemental Figure 2.png”): Plot of the clinician-provided frailty ratings against the total word count in the eConsult text. FR, frailty rating.

**SUPPLEMENTAL METHODS**

**Patient and Public Involvement**

A multidisciplinary team co-designed this study using an integrated knowledge translation approach.(45) Stakeholders and knowledge users (i.e., clinicians and patient partners) have been involved in all stages of the study, including the development of the research questions, the modification of the list of frailty-related terms, and the interpretation of results.

**Clinician-led review of frailty content on eConsult**

Before beginning the case review, the clinicians and other members of the research team met to discuss the frailty rating task and achieve consensus on how the cases should be assessed. Through discussion, an explicit distinction was made between the Clinical Frailty Scale (i.e., a clinical tool used to rate the degree of frailty on a spectrum) (46), and the frailty rating in the present study, which specifically serves to estimate the likelihood that a patient is living with any degree of frailty (i.e., an assessment ranging from a low to high likelihood that a patient is living with frailty based on the available eConsult data). Clinicians also provided feedback regarding the frailty rating task, resulting in an additional coding option being added to indicate that insufficient information was available in the eConsult case to make a judgement about frailty (i.e., “Not enough information to make a frailty rating.”).

**Phase 1 – Preparing a key-term search to identify frailty content in eConsult**

The working group of clinicians, researchers, and a patient partner were consulted for feedback by drawing from the discussion-generating questions posed in Urquhart et al.’s rule development strategy (23). Discussants were asked whether they agreed or disagreed with the selected terms, whether any important terms or concepts should be added, and about their views on the feasibility of searching for these terms or phrases in the provider-to-provider communication captured on eConsult. Suggestions from the working group were used to update the list.

**Phase 2 – Developing an eConsult text-searching computer algorithm**

The following Python libraries were used to develop the text-parsing computer algorithm: NumPy, pandas, xlrd, csv, string, TextBlob, and nltk.

Data cleaning and preparation were performed for the list of frailty-related terms (collated in Phase 1) and the eConsult text to make them amenable as inputs into the text-parsing Python algorithm.

For the list of frailty-related terms, this included amalgamating findings from various sources of frailty-related terms identified in Phase 1 and applying a uniform format, removing any references to healthcare data items not applicable to eConsult data (e.g., diagnostic codes), removing duplicates, removing unnecessary punctuation, lowering the case of all letters, increasing the generalizability of the terms/phrases (e.g., by removing references to specific patient circumstances), and removing subjects or pronouns at the beginning of phrases. The terms/phrases were organized into a tab-separated values (TSV) format for input into the text parsing algorithm.

The eConsult text was derived from communication logs between PCPs and specialists interacting through the eConsult platform. The communication logs, comprising the initial query from the PCP to the specialist, the specialist’s response, and any other ensuing interactions, were extracted and organized into TSV format. Additionally, when users of the eConsult platform attached additional material to the eConsult in the form of additional text-based information (i.e., not imaging or lab reports) that was relevant to the case, this was appended to the communication logs.

**Supplemental Table S2**. Number of cases and mean overall word count per eConsult case, stratified by the number of frailty-related terms identified in the complete eConsult communication logs.

| **Frequency of frailty-related terms identified (n)** | **Number of cases (n)** | **Overall word count (mean ± SD)** |
| --- | --- | --- |
| 0 | 52 | 378.33 ± 442.19 |
| 1 | 41 | 469.32 ± 243.26 |
| 2 | 26 | 457.77 ± 188.09 |
| 3 | 26 | 594.58 ± 443.13 |
| 4 | 17 | 528.29 ± 247.84 |
| 5 | 15 | 594.53 ± 245.89 |
| 6 | 12 | 616.08 ± 257.23 |
| 7 | 9 | 513.67 ± 159.10 |
| 8 | 10 | 603.80 ± 292.38 |
| 9 | 4 | 657.50 ± 358.80 |
| 10 | 4 | 613.25 ± 125.54 |
| >10 | 9 | 1144.00 ± 829.07 |
| Total sample | 225 | 522.70 ± 383.05 |
