# Supplementary figures and images for: Using electronic consultation (eConsult) to identify frailty in provider-to-provider communication: a feasibility and validation study
Source: BMC Geriatr. 2023 Mar 9;23:136. doi: 10.1186/s12877-023-03870-w (PMC9999527; doi:10.1186/s12877-023-03870-w)

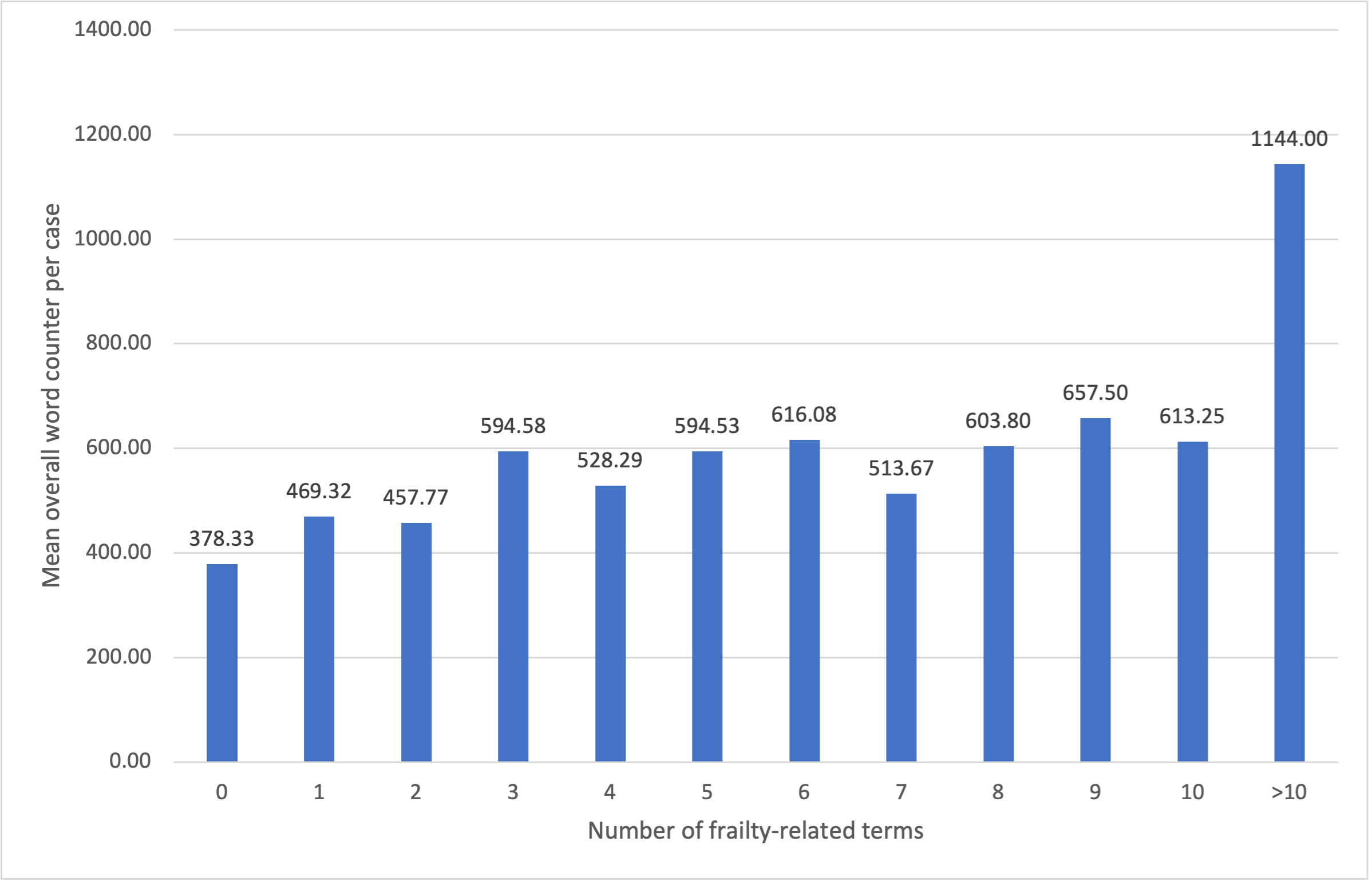

Supplement: Supplementary file 1 — Additional file 1: Supplemental Methods: a description of the methods for patient and public involvement, clinician-led review of frailty related content on eConsult, and phase 1-2 of developing a frailty identification approach using eConsult. Supplemental Table S1. List of frailty-related terms for each frailty topic. List of frailty-related terms for each of the 17 topics. Supplemental Table S2. Prevalence of eConsult cases stratified by number of frailty-related terms. Number of cases and mean overall word count per eConsult case, stratified by the number of frailty-related terms identified in the complete eConsult communication logs. Supplemental Figure S1. Mean overall word count per eConsult case, stratified by the number of frailty-related terms identified in the complete eConsult communication log. Supplemental Figure S2. Plot of the clinician-provided frailty ratings against the total word count in the eConsult text. [file 12877_2023_3870_MOESM1_ESM.zip › Supplemental Figure 1.png]

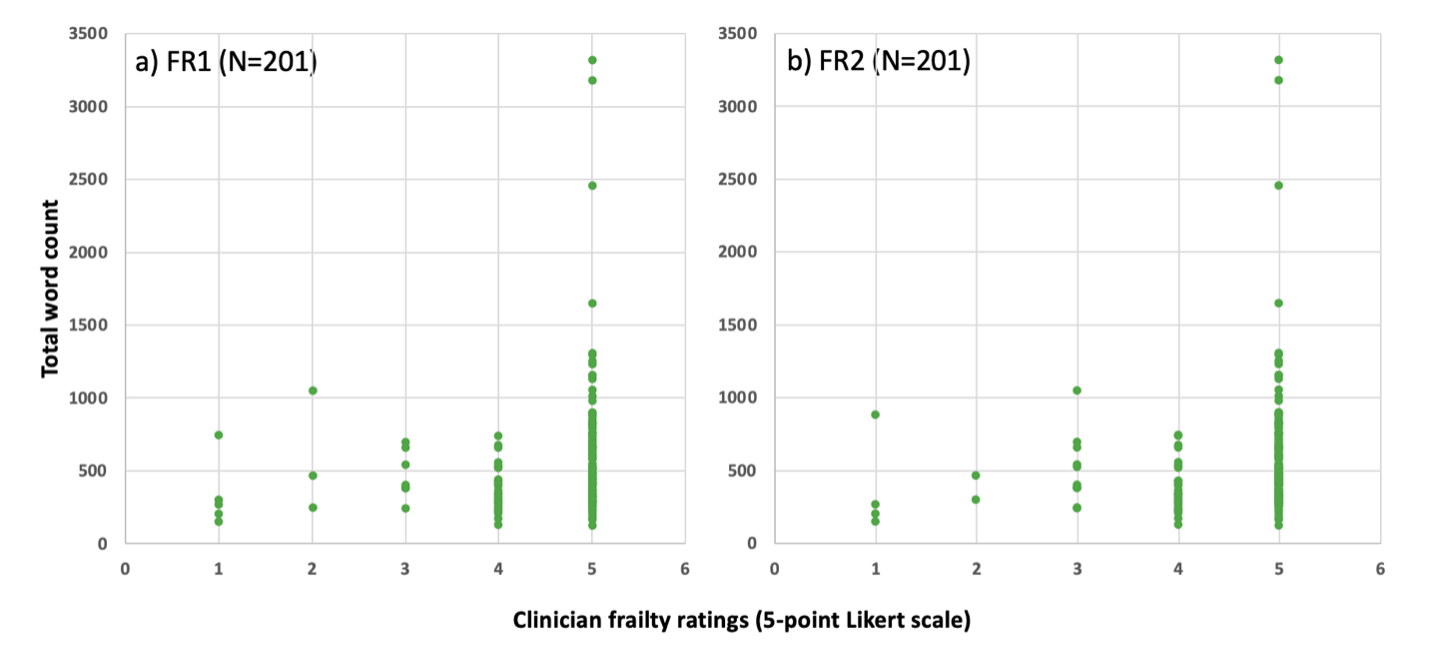

Supplement: Supplementary file 1 — Additional file 1: Supplemental Methods: a description of the methods for patient and public involvement, clinician-led review of frailty related content on eConsult, and phase 1-2 of developing a frailty identification approach using eConsult. Supplemental Table S1. List of frailty-related terms for each frailty topic. List of frailty-related terms for each of the 17 topics. Supplemental Table S2. Prevalence of eConsult cases stratified by number of frailty-related terms. Number of cases and mean overall word count per eConsult case, stratified by the number of frailty-related terms identified in the complete eConsult communication logs. Supplemental Figure S1. Mean overall word count per eConsult case, stratified by the number of frailty-related terms identified in the complete eConsult communication log. Supplemental Figure S2. Plot of the clinician-provided frailty ratings against the total word count in the eConsult text. [file 12877_2023_3870_MOESM1_ESM.zip › Supplemental Figure 2.png]
